# Anatomical and Systemic Risk Factors for Recurrence in Medication-Related Osteonecrosis of the Jaw (MRONJ): A Retrospective Study of 812 Patients

**DOI:** 10.3390/jcm15134936

**Published:** 2026-06-25

**Authors:** Kyoung-Chan Park, Hyo-Joon Kim, Ji-Su Oh, Seong-Yong Moon

**Affiliations:** 1 Department of Oral and Maxillofacial Surgery, Chosun University Dental Hospital, Gwangju 61452, Republic of Korea; goodchanny@chosun.kr (K.-C.P.); hyojoonkim@chosun.ac.kr (H.-J.K.); jsoh@chosun.ac.kr (J.-S.O.); 2 Department of Oral and Maxillofacial Surgery, College of Dentistry, Chosun University, Gwangju 61452, Republic of Korea

**Keywords:** medication-related osteonecrosis of the jaw, recurrence, risk factors, mandibular ramus, liver diseases

## Abstract

**Background/Objectives**: Medication-related osteonecrosis of the jaw (MRONJ) is a severe complication of antiresorptive and antiangiogenic therapies, and identifying specific risk factors for recurrence remains a significant clinical challenge. This study aimed to evaluate the clinical characteristics and independent risk factors for recurrence in a large-scale cohort of MRONJ patients. **Methods**: This single-center retrospective study analyzed 812 patients diagnosed with MRONJ according to the 2022 AAOMS criteria at Chosun University Dental Hospital between 2020 and 2024. Demographic, clinical, radiographic, and medication-related variables were collected. Univariate and multivariate logistic regression analyses were performed to identify independent risk factors associated with recurrence. **Results**: The majority of patients were female (89.9%), with a mean age of 72.9 years, and mandibular involvement was most frequent (70.8%). Tooth extraction was the most common local precipitating factor (47.0%). The overall recurrence rate was 10.1%. Multivariate analysis identified bilateral jaw involvement (OR = 4.555, *p* = 0.006), mandibular ramus involvement (OR = 8.222, *p* = 0.008), and systemic liver disease (OR = 3.703, *p* = 0.037) as significant independent risk factors. Conversely, routes of prior antiresorptive medication administration involving intravenous-only or combined oral/intravenous therapy, as well as hyperlipidemia and a history of dental implant surgery, were associated with lower recurrence rates. **Conclusions**: Anatomical complexity and systemic health status are critical predictors of MRONJ recurrence. Patients presenting with bilateral or mandibular ramus involvement, or with compromised liver function, require more aggressive surgical debridement and individualized treatment planning to reduce the risk of recurrence. Given the small affected subgroups, the effect sizes for mandibular ramus involvement and liver disease should be interpreted with caution.

## 1. Introduction

Medication-related osteonecrosis of the jaw (MRONJ) is a severe and debilitating adverse drug reaction characterized by progressive bone destruction in the maxillofacial region of patients exposed to antiresorptive or antiangiogenic agents [1,2]. While classical chronic suppurative osteomyelitis (CSO) is primarily an inflammatory condition driven by microbial infection within the medullary cavity, MRONJ represents a distinct disease entity with a complex pathophysiology involving suppressed bone remodeling and inhibition of angiogenesis [3,4]. Since its first clinical description, the incidence of MRONJ has risen significantly due to the widespread use of bisphosphonates and denosumab for managing osteoporosis and bone metastases [5,6].

The 2022 American Association of Oral and Maxillofacial Surgeons (AAOMS) update has refined the diagnostic criteria, yet the management of advanced-stage MRONJ remains a formidable challenge for clinicians. Stage 1 MRONJ is characterized by exposed and necrotic bone, or a fistula that probes to bone, in asymptomatic patients without evidence of infection. Stage 2 disease presents with exposed and necrotic bone associated with clinical signs of infection, including pain, erythema, and purulent drainage. Stage 3 MRONJ represents advanced disease with exposed necrotic bone and infection accompanied by at least one additional complication, such as extension beyond the alveolar bone region, pathologic fracture, extraoral fistula, oroantral communication, or osteolysis extending to the inferior border of the mandible or the sinus floor [5]. The pathophysiology of MRONJ is heavily intertwined with altered immune surveillance and local inflammatory amplification. Antiresorptive overdosage not only inhibits osteoclastic functions but also triggers a profound suppression of local angiogenesis and disrupts T-cell-mediated immune responses, significantly exacerbating micro-environmental susceptibility to micro-trauma and infectious challenges. Recent literature emphasizes that surgical outcomes in MRONJ are predominantly dependent on the thoroughness of the surgical technique and the complete elimination of all necrotic foci [7,8]. Although surgical intervention is increasingly recognized as a definitive treatment for Stage 2 and 3 disease, postoperative recurrence frequently complicates the clinical course [9,10]. Previous studies have explored various systemic and local factors, yet the specific predictors of recurrence in large-scale clinical cohorts remain insufficiently characterized [11,12].

Anatomical complexities, such as involvement of the mandibular ramus or bilateral lesions, may pose technical difficulties during surgical debridement, potentially leading to incomplete removal of necrotic bone [13]. Furthermore, the impact of systemic comorbidities like liver disease on the regenerative capacity of jawbone tissue is an area that requires deeper investigation, as impaired healing environments can significantly increase the risk of surgical failure [14,15]. In aging populations, the clinical burden of MRONJ continues to escalate, necessitating a more refined understanding of its incidence and cure rates [16].

Therefore, this single-center retrospective study was designed to analyze a substantial cohort of 812 MRONJ patients in Chosun University. By focusing exclusively on MRONJ and identifying independent risk factors associated with recurrence, this study aims to provide robust clinical evidence to guide surgical decision-making and improve long-term treatment outcomes.

## 2. Materials and Methods

### 2.1. Patient Selection and Inclusion/Exclusion Criteria

This single-center retrospective study was conducted in accordance with the Declaration of Helsinki and approved by the Institutional Review Board (IRB). We initially identified a total of 1246 patients diagnosed with inflammatory jaw diseases at Chosun University Dental Hospital between January 2020 and December 2024. However, to address concerns regarding the heterogeneity of osteomyelitis subtypes and to ensure statistical validity, we implemented a rigorous selection process to focus exclusively on medication-related osteonecrosis of the jaw (MRONJ) [1,4].

Patients were included in the final analysis if they met the following criteria based on the 2022 AAOMS update: (1) current or previous treatment with antiresorptive or antiangiogenic agents; (2) exposed bone or bone that can be probed through an intraoral or extraoral fistula in the maxillofacial region persisting for more than 8 weeks; and (3) no history of radiation therapy or metastatic disease to the jaws [5,16]. To maintain data integrity and focus on surgical outcomes, we excluded 434 patients who were diagnosed with conventional chronic suppurative osteomyelitis (CSO), mixed-type osteomyelitis, or those who had incomplete clinical records and a follow-up period of less than 6 months. Consequently, a total of 812 MRONJ patients were included in the final cohort for analysis.

### 2.2. Standardized Surgical Protocol and Operational Definition of Recurrence

The clinical management strategy for the 812 enrolled MRONJ patients was determined based on the severity of symptoms, baseline AAOMS staging, and individual systemic tolerances. Conservative management, consisting strictly of long-term empirical antibiotic therapy and intensive topical chlorhexidine rinses, was preferentially indicated for patients demonstrating asymptomatic Stage 1 stability or those with temporary systemic contraindications to general anesthesia. Conversely, definitive surgical intervention was aggressively indicated for patients presenting with advanced-stage disease (Stages 2 and 3), persistent clinical symptoms (e.g., secondary infection, refractory pain), or radiographic evidence of progressive bone sequestration that failed to respond to initial conservative measures.

Of the total cohort, 767 patients underwent standardized surgical interventions performed by experienced oral and maxillofacial surgeons at our institution. The primary surgical protocol consisted of thorough surgical debridement or sequestrectomy. Under preoperative cone-beam computed tomography (CBCT) and clinical guidance, aggressive removal of all non-viable, necrotic bone was performed until fresh, bleeding vital bone margins were reached. Following complete elimination of the necrotic foci, peripheral ostectomy was performed where necessary to secure healthy bone margins, and primary mucosal closure was achieved whenever feasible using layered suturing techniques. Postoperatively, all patients received empirical antibiotic therapy and chlorhexidine mouth rinses according to institutional guidelines. Surgical modality was selected according to lesion extent: sequestrectomy for localized, well-demarcated lesions; saucerization/decortication for broader, poorly demarcated necrotic margins; and segmental or marginal resection for extensive Stage 3 disease. Intraoperatively, the viable surgical margin was defined by uniform punctate bleeding from both the cortical plates and the medullary cavity.

Following initial surgical healing, patients were routinely monitored at 1 week, 1 month, 3 months, and every 3 to 6 months thereafter. MRONJ recurrence was operationally defined as the re-emergence of clinical bone exposure, persistent non-healing fistulas, or radiographic evidence of progressive bone osteolysis at the prior surgical site after achieving complete mucosal healing post-surgery. At each visit, a comprehensive clinical examination—visual inspection and digital palpation to assess mucosal coverage, bone exposure, fistula formation, and signs of secondary infection—was performed by senior oral and maxillofacial surgeons, supplemented by panoramic radiography and, when recurrence was clinically suspected, CBCT to detect new or expanding osteolysis, sequestrum formation, or cortical bone defects.

### 2.3. Clinical Variables and Statistical Analysis

Clinical variables collected for these 812 patients included demographics (sex, age), MRONJ staging, and detailed medication history [6]. Systemic conditions such as hypertension, diabetes, and liver disease, as well as local etiologic factors like tooth extraction and dental implant surgery, were thoroughly evaluated [14,16]. Radiographic assessment was performed CBCT to identify specific hard tissue changes, including sequestrum formation and cortical bone defects, which are critical for determining surgical margins [17,18]. For the anatomical distribution analysis in Table 1, in cases where both the maxilla and mandible were involved, the primary site was determined based on the region with more extensive clinical or radiographic destruction. The term ‘both jaws’ was operationally defined as the simultaneous or sequential development of distinct MRONJ lesions in both the maxilla and mandible within the same patient, rather than a single crossing lesion. The phrase ‘anatomical complexity’ specifically refers to regions presenting restricted surgical access, dense cortical bone thickness with poor vascularity (such as the mandibular ramus), or multi-focal involvements compromising primary soft tissue closure. All radiographic assessments were performed using cone-beam computed tomography (CBCT; Planmeca Viso G7, Planmeca Oy, Helsinki, Finland) and panoramic radiography (Planmeca ProMax, Planmeca Oy, Helsinki, Finland).

Statistical analysis was performed using IBM SPSS Statistics, version 26.0 (IBM Corp., Armonk, NY, USA). Descriptive statistics were used to summarize the clinical characteristics of the 812 patients. Univariate and multivariate logistic regression analyses were conducted to identify independent risk factors for MRONJ recurrence, with the significance level set at *p* < 0.05 [9,10]. Univariate analysis utilized the Chi-square test or Student’s *t*-test to compare clinical variables according to recurrence status. Subsequently, multivariate logistic regression analysis was performed, adjusting for potential baseline confounders including MRONJ staging. Candidate variables for the multivariate model were selected based on clinical relevance and a univariate significance level of *p* < 0.05. The model’s goodness-of-fit was evaluated using the Hosmer–Lemeshow test.

## 3. Results

### 3.1. Demographic and Clinical Characteristics

Among the total of 812 patients included in the final analysis, 730 (89.9%) were female and 82 (10.1%) were male, with a mean age of 72.9 years. Table 1 summarizes the anatomical distribution of the lesions on CT images. The mandible was the most frequent site of involvement (70.8%, *n* = 575), with the mandibular body being the most common subregion (61.8%, *n* = 502). Maxillary lesions accounted for 29.2% of the cases (*n* = 237), primarily involving the posterior region (20.3%, *n* = 165). Radiographic assessment of hard tissue changes (Table 2) showed that sequestrum formation was the most frequent finding (43.6%, *n* = 354), followed by cortical bone defects (37.6%, *n* = 305). Regarding the baseline severity of the disease, 216 patients (26.6%) were classified as Stage 1, 511 (62.9%) as Stage 2, and 85 (10.5%) as Stage 3 (Table 1). Regarding the clinical management and treatment modalities of the 812 included patients, the vast majority underwent definitive surgical interventions (*n* = 767, 94.5%), predominantly consisting of sequestrectomy (*n* = 647, 79.7%) and saucerization or decortication (*n* = 112, 13.8%), while a minor subset received extensive mandibulectomy (*n* = 8, 1.0%). In contrast, 45 patients (5.5%) who presented with transient contraindications to surgery or early-stage stability were managed strictly via conservative antibiotic therapy and intensive topical rinses (Table 3).

Systemic comorbidities and local etiologic factors were also evaluated at baseline. Hypertension was the most common systemic condition (60.0%, *n* = 487), followed by diabetes mellitus (28.4%, *n* = 231) and hyperlipidemia (17.6%, *n* = 143) (Table 4). Regarding local etiologic factors, tooth extraction was the leading precipitating event (47.0%, *n* = 382), followed by dental implant surgery (23.1%, *n* = 188) and spontaneous odontogenic infection (12.3%, *n* = 100), as summarized in Table 5.

### 3.2. Factors Associated with Recurrence

The overall recurrence rate in the study population was 10.1% (82/812 patients). Univariate analysis identified several factors significantly associated with higher recurrence rates (Table 6). Unlike the anatomical distribution in Table 1, patients with involvement of both jaws were analyzed as a distinct group (‘Both jaw’) in Table 6 to evaluate the impact of bilateral lesions on recurrence. Bilateral jaw involvement showed a significantly higher recurrence rate (25.9%) compared to unilateral maxillary (8.8%) and mandibular (9.3%) involvement (*p* = 0.021). The oral route of drug administration also showed a significantly higher recurrence rate (14.8%) compared to intravenous (7.5%) or combined routes (4.9%) (*p* = 0.001). Regarding the 27 patients with bilateral jaw involvement analyzed in Table 6, these individuals were initially cross-classified into their respective primary lesion sites in Table 1 based on the location of more extensive bone destruction, ensuring that the total cohort size (*N* = 812) remained consistent across all baselines. The median postoperative follow-up duration was 18.4 months (range, 6.0–48.0 months), with all included patients observed for a minimum of 6 months. Recurrence rates differed across surgical modalities, occurring in 9.4% (61/647) of sequestrectomy cases, 14.3% (16/112) of saucerization/decortication cases, and 0% (0/8) of major resection cases, while 11.1% (5/45) of conservatively managed patients experienced recurrence (Table 3).

Regarding the temporal onset of recurrence, the mean time to recurrence was 5.2 ± 2.4 months postoperatively (range, 1.5–14.0 months). Early-onset recurrence within 3 months occurred in 20.7% (17/82) of cases, the majority (58.5%, 48/82) appeared between 3 and 6 months, and the remaining 20.8% (17/82) manifested after 6 months, underscoring the need for continued long-term surveillance.

When evaluating systemic conditions according to recurrence status via univariate analysis (Table 4), liver disease was significantly associated with a high recurrence rate of 25.0% (*p* = 0.046). Conversely, hyperlipidemia (4.9%, *p* = 0.023) and the use of anticoagulants (3.0%, *p* = 0.044) were significantly associated with lower recurrence rates. Other systemic comorbidities, such as hypertension, diabetes mellitus, and chronic kidney disease, did not demonstrate statistically significant relationships with disease recurrence (*p* > 0.05).

Anatomical subregion locations and specific radiographic findings were further cross-tabulated with recurrence status to identify local indicators of treatment failure (Table 7). Lesions located in the mandibular ramus (30.0%, *p* = 0.036) and the posterior maxilla (14.5%, *p* = 0.034) were significantly linked to increased recurrence risks. In contrast, specific hard tissue changes observed on CT images—including defect in the trabecular bone, osteosclerosis, defect in the cortical bone, sequestrum formation, and periosteal reaction—did not show statistically significant differences in recurrence rates between the groups (*p* > 0.05).

### 3.3. Multivariate Logistic Regression Analysis

Multivariate logistic regression analysis was performed to identify independent predictors of MRONJ recurrence, simultaneously adjusting for baseline clinical factors and MRONJ staging (Table 8). Even after controlling for disease severity, anatomical location and systemic health status remained critical predictors of treatment failure.

Bilateral jaw involvement conferred a 4.5-fold increased risk of recurrence compared to unilateral maxillary involvement (OR = 4.555, 95% CI: 1.542–13.46, *p* = 0.006). Anatomically, mandibular ramus involvement was significantly associated with an increased risk of recurrence (OR = 8.222, 95% CI = 1.745–38.740, *p* = 0.008). However, given the limited number of affected cases and the wide confidence interval, the magnitude of this association should be interpreted with caution. Systemic liver disease was also identified as a significant independent risk factor (OR = 3.703, 95% CI: 1.079–12.711, *p* = 0.037), although it exhibited a wide confidence interval. Notably, baseline MRONJ staging demonstrated a strong, dose-dependent independent association with recurrence. Compared to Stage 1 disease, patients with Stage 2 disease showed a 2.1-fold increase in recurrence risk (OR = 2.104, 95% CI: 1.145–3.864, *p* = 0.017), while those with Stage 3 disease exhibited a 5.5-fold higher risk of treatment failure (OR = 5.485, 95% CI: 2.314–13.012, *p* < 0.001).

Factors significantly associated with a reduced risk of recurrence included intravenous drug administration (OR = 0.501, *p* = 0.015), combined oral and IV administration (OR = 0.281, *p* = 0.002), hyperlipidemia (OR = 0.398, *p* = 0.034), and prior dental implant surgery (OR = 0.318, *p* = 0.004). To assess the statistical reliability of the final multivariate model given the 82 recurrence events, the events-per-variable (EPV) ratio was maintained above acceptable methodological thresholds to avoid overfitting. The model demonstrated excellent goodness-of-fit, as indicated by the Hosmer–Lemeshow test (χ^2^ = 7.412, *p* = 0.493) and a robust area under the receiver operating characteristic curve (ROC-AUC = 0.814), confirming its strong predictive accuracy.

## 4. Discussion

This study analyzed a large-scale cohort of 812 MRONJ patients to identify independent risk factors for recurrence following surgical intervention. By focusing exclusively on MRONJ and excluding conventional osteomyelitis cases, we aimed to provide clearer clinical evidence for this distinct disease entity. Our findings emphasize that both anatomical complexities and systemic patient factors significantly influence surgical outcomes, echoing previous clinical evaluations regarding post-surgical prognosis and risk distribution [19].

A key finding of this study is the critical role of anatomical location in predicting recurrence. Specifically, lesions involving the mandibular ramus and bilateral jaw involvement were associated with significantly higher risks of treatment failure (OR 8.222 and 4.555, respectively). These results align with recent literature emphasizing that surgical success in MRONJ is heavily dependent on the thoroughness of the surgical technique and the complete elimination of all necrotic bone [7,8]. As noted by Voss et al., achieving clean surgical margins in anatomically challenging areas like the ramus can be technically demanding, potentially leading to incomplete debridement [7]. Furthermore, Ristow et al. highlighted that surgical intervention is often more effective than conservative management for advanced stages, provided that a radical approach is adopted to ensure the removal of all infected foci [8].

This anatomical vulnerability is closely linked to our standardized surgical protocol, which mandates aggressive removal of non-viable bone until fresh, bleeding vital margins are identified. In lesions localized to the mandibular ramus, achieving this biological endpoint presents a formidable technical challenge. The mandibular ramus presents substantial surgical challenges because of its limited accessibility, thick cortical bone, and difficulty in defining viable bleeding margins intraoperatively [19]. Consequently, microscopic foci of necrotic bone or bacterial bio-films may inadvertently remain within the marrow spaces, acting as a nidus for post-surgical osteolysis and subsequent clinical recurrence (OR = 8.222). This extreme hazard magnitude (OR = 8.222) demonstrates a stark contrast to general lower jaw risk allocations in the established literature. For instance, Klingelhöffer et al. allocated a considerably lower recurrence risk parameter (OR = 2.15) when evaluating general mandibular posterior boundaries. The distinct escalation in our reported effect size is heavily justified by our precise isolation of the ‘ramus’ as an independent anatomical entity. While prior studies diluted the risk by clustering the whole mandible or posterior arches, our focused segregation exposes the true, uncompromised biological severity imposed by the thick cortical density and extreme restriction of direct visual access inherent to the ramus anatomy [19].

Furthermore, the clinical management and treatment modalities of our cohort provide crucial context for interpreting these recurrence dynamics. To maintain data integrity and strictly evaluate post-surgical outcomes, our patient selection protocol specifically focused on individuals who underwent definitive surgical interventions—predominantly surgical debridement and sequestrectomy (*n* = 767, 94.5%)—rather than long-term conservative management. This programmatic focus on surgical therapy aligns with the expanding clinical consensus that active surgical elimination of necrotic foci is far superior to conservative strategies for resolving advanced-stage MRONJ [7,8,20,21]. By establishing a highly standardized, surgery-centric cohort, we minimized the potential confounding variations introduced by mixed treatment modalities. However, as noted by the reviewers, this approach inherently demands that ‘recurrence’ be framed strictly as a surgical treatment failure rather than a biological progression under conservative monitoring. The thoroughness of the initial operational approach remains a primary clinical determinant; even within a single-center protocol where surgical techniques are heavily cross-standardized, microscopic margins can fluctuate, heavily highlighting why extensive baseline necrotic burden serves as a primary driving force behind subsequent operations.

Similarly, the high recurrence risk observed in patients with bilateral jaw involvement (OR = 4.555) highlights the aggressive and systemic nature of their disease manifestation. Bilateral presentation typically reflects a prolonged duration of high-potency antiresorptive exposure or a highly compromised systemic bone remodeling apparatus. When executing sequential or simultaneous bilateral surgeries, maintaining architectural stability while striving for complete debridement often limits the surgical radicality. Furthermore, securing tension-free primary mucosal closure—a pivotal component of our institutional protocol—is exceptionally difficult in bilateral sites due to scarred, retracted soft tissue beds. Failure to maintain a stable soft tissue seal postoperatively inevitably leads to premature wound dehiscence and bacterial re-contamination of the underlying bone, explaining the nearly 4.5-fold increase in treatment failure in this specific subgroup. When critically evaluated against previous reports, the effect size observed in our cohort (OR = 4.555) is profoundly higher than those reported in the literature, such as the matched multicenter analysis by Hayashida et al. (OR = 2.45). This pronounced variance in effect size highlights the structural and administrative advantage of our single-center protocol, which enforced a meticulous, longitudinal time-to-event tracking mechanism (median 18.4 months). This allowed for the comprehensive capture of sequential contralateral recurrences that are frequently under-reported or misclassified as single persistent lesions in more heterogeneous multi-center registries [9].

Regarding systemic factors, liver disease was identified as a significant independent risk factor for recurrence (OR 3.703). However, the relatively small number of patients with liver disease (*n* = 16) in our cohort represents a notable limitation. While the statistical significance (*p* = 0.037) suggests a strong association, the wide confidence interval (1.079–12.711) indicates that these results should be interpreted with caution. Nevertheless, impaired bone metabolism and reduced regenerative capacity in patients with chronic liver disease may contribute to poorer healing environments, increasing the risk of surgical failure [14,15,22]. It is worth noting that while classical osteology updates by Guañabens et al. primarily concentrated on the broad systemic correlation between hepatic cirrhosis and general bone mineral density degradation, our study is among the first to establish a concrete, independent clinical outcome risk size (OR = 3.703) linking liver compromise directly to post-operative surgical recurrence. This critical comparison shifts the clinical narrative from theoretical bone frailty to an empirical hazard index, providing a rigorous benchmark for perioperative patient optimization [22].

Interestingly, hyperlipidemia and prior dental implant surgery were associated with lower recurrence rates. The protective effect of hyperlipidemia might be indirectly linked to the use of statins, which have been suggested in some studies to promote bone formation and reduce inflammation, although further prospective research is required to confirm this mechanism. The lower recurrence rate in the implant group should be interpreted cautiously as a hypothetical manifestation of the ‘healthy patient effect’ rather than a direct biological mechanism. Individuals cleared for implant surgeries typically present with superior baseline systemic reserves and optimal oral hygiene compliances, which may indirectly favor mucosal healing. However, the precise underpinnings remain elusive, and further clinical validation is required.

Nevertheless, the mechanisms underlying the observed associations between hyperlipidemia, prior implant surgery, routes of antiresorptive medication administration, and MRONJ recurrence remain uncertain. Although several biological and clinical explanations have been proposed in the literature, the present retrospective study was not designed to establish causal relationships. Therefore, these findings should be interpreted as statistical associations rather than evidence of direct mechanistic effects, and further prospective studies are required to clarify the underlying pathways.

Furthermore, our multivariate analysis revealed a lower recurrence rate among patients with intravenous (IV) drug administration compared to those on oral medications, a finding that initially appears counterintuitive given the higher potency of IV antiresorptives. Rather than a true biological protective effect, this paradox is highly likely a reflection of informative censoring and competing risks. A significant portion of IV patients in our cohort were oncologic individuals who typically exhibit shorter overall survival and a higher mortality rate due to their underlying malignancies [23]. Consequently, these high-risk patients may have experienced informative censoring—where their follow-up time was prematurely truncated before post-surgical recurrence could be clinically observed or documented. This competing risk of mortality should be carefully considered when evaluating the impact of administration routes on long-term surgical prognosis.

Despite the clinical significance of our findings, several important limitations inherent to this study must be explicitly acknowledged. First, this investigation was designed as a single-center retrospective study conducted exclusively at a university dental hospital in the Republic of Korea. Consequently, the study population reflects a specific regional and demographic homogeneity, which raises valid questions regarding the immediate generalizability of our results to more ethnically and geographically diverse global populations. The retrospective nature of the data collection also depends entirely on historical medical records, which introduces a potential risk of selection bias or missing confounding variables that could not be accounted for in our statistical models.

Second, our cohort exhibits a stark sex distribution imbalance, with female patients comprising 89.9% (*n* = 730) of the total 812 participants. While this high proportion accurately mirrors the epidemiological reality of osteoporosis management in aging East Asian populations—where the vast majority of patients prescribed long-term antiresorptive agents are postmenopausal females—it heavily underrepresents male patients (10.1%, *n* = 82). This severe demographic skew limits our ability to evaluate sex-specific physiological interactions with MRONJ recurrence risks.

Furthermore, as previously noted, the statistical power of certain specific systemic and local subgroups—such as patients with liver disease (*n* = 16) or mandibular ramus lesions (*n* = 10)—remains statistically fragile, resulting in wide 95% confidence intervals that demand a highly conservative interpretation of their exact clinical effect sizes. To overcome these single-center constraints, future research must implement large-scale, prospective, multi-center studies utilizing diverse, multi-ethnic patient cohorts to firmly establish and validate these predictive risk models.

## 5. Conclusions

In conclusion, this large-scale retrospective study demonstrates that specific anatomical configurations—namely bilateral jaw involvement and lesions involving the mandibular ramus—significantly elevate the risk of postoperative MRONJ recurrence. Furthermore, advanced baseline MRONJ staging (Stages 2 and 3) carries a powerful, dose-dependent risk of treatment failure, whereas systemic conditions like liver disease serve as critical indicators of compromised bone healing environments.

Clinicians must prioritize a meticulous and radical surgical approach aimed at the aggressive elimination of all necrotic foci and securing stable, tension-free primary mucosal closure, particularly when managing these high-risk subgroups. Intensive postoperative monitoring and individualized perioperative care are highly recommended to minimize the clinical burden of re-operation and maximize long-term treatment success.

## Figures and Tables

**Table 1:** Anatomical distribution and clinical staging of MRONJ on CBCT images (*N* = 812).

Variable	Subcategory	*N* (%)
Anatomical distribution		
Maxilla (*n* = 237, 29.2%)	Anterior	72 (8.9)
	Posterior	165 (20.3)
Mandible (*n* = 575, 70.8%)	Incisal	53 (6.5)
	Body	502 (61.8)
	Angle	9 (1.1)
	Ramus	10 (1.2)
	Condyle	1 (0.1)
MRONJ staging	Stage 1	216 (26.6)
	Stage 2	511 (62.9)
	Stage 3	85 (10.5)

Note: For the anatomical distribution (Table 1), cases involving both the maxilla and mandible were categorized based on the site with more extensive destruction.

**Table 2:** Radiographic findings of hard tissue changes on CBCT images (*N* = 812).

Hard Tissue Changes	*N* (%)
Defect in the trabecular bone	97 (11.9)
Osteosclerosis	34 (4.2)
Defect in the cortical bone	305 (37.6)
Sequestrum	354 (43.6)
Periosteal reaction	22 (2.7)

**Table 3:** Distribution of initial treatment modalities among the evaluated cohort (*N* = 812).

Treatment Modality	Number of Patients (*n*)	%	Recurrence, *n* (%)
Surgical Management	767	94.5	77 (10.0)
Sequestrectomy	647	79.7	61 (9.4)
Saucerization/Decortication	112	13.8	16 (14.3)
Mandibulectomy or Major Resection	8	1.0	0 (0.0)
Conservative Management (Antibiotics only)	45	5.5	5 (11.1)

**Table 4:** Univariate Comparisons of Systemic Conditions Between the Recurrence and Non-Recurrence Groups (*N* = 812).

Variable	*N*	Recurrence (%)	χ^2^ or t	*p*-Value
Hypertension	487	52 (10.7)	0.449	0.503
Hyperlipidemia	143	7 (4.9)	5.176	0.023
Diabetes mellitus	231	21 (9.1)	0.361	0.548
Taking anticoagulants	67	2 (3)	4.070	0.044
Heart disease	100	5 (5)	3.265	0.071
Thyroid disease	27	3 (11.1)	0.032	0.859
Liver disease	16	4 (25)	3.992	0.046
Kidney disease	18	1 (5.6)	0.418	0.518
Rheumatoid arthritis	46	6 (13)	0.466	0.495
Cancer	65	7 (10.8)	0.035	0.852
Dementia	57	2 (3.5)	2.932	0.087
Steroids	8	1 (12.5)	0.051	0.821
Chemotherapy	13	3 (23.1)	2.451	0.117
Other immune-related diseases	5	0 (0)	0.565	0.452

**Table 5:** Univariate Comparisons of Local Etiologic Factors Between the Recurrence and Non-Recurrence Groups (*N* = 812).

Variable	*N*	Recurrence (%)	χ^2^ or t	*p*-Value
Extraction	382	45 (11.8)	2.247	0.134
Trauma	42	4 (9.5)	0.016	0.899
Implant surgery	188	8 (4.3)	9.200	0.002
Bone surgery	10	1 (10)	0.01	0.922
Dental origin infection	100	14 (14)	1.912	0.167

**Table 6:** Univariate Comparisons of Demographic and Clinical Variables Between the Recurrence and Non-Recurrence Groups (*N* = 812).

Variable	*N*	Recurrence (%)	χ^2^ or t	*p*-Value
Sex				
Male	82	11 (13.4)	1.105	0.293
Female	730	71 (9.7)		
Lesion site				
Maxilla	237	21 (8.8)	7.769	0.021
Mandible	575	54 (9.3)		
Both jaw	27	7 (25.9)		
Route of drug administration				
Oral	345	51 (14.8)	15.28	0.001
IV	305	23 (7.5)		
Both	162	8 (4.9)		

**Table 7:** Univariate Comparisons of Anatomical Lesion Locations and Radiographic Findings Between the Recurrence and Non-Recurrence Groups (*N* = 812).

Variable	*N*	Recurrence (%)	χ^2^ or t	*p*-Value
**<Affected area>**				
Maxilla—Ant. area	72	6 (8.2)	0.312	0.576
Maxilla—Post. area	165	24 (14.5)	4.511	0.034
Mandible—Incisal area	53	4 (7.5)	0.407	0.524
Mandible—Body	502	45 (9)	1.864	0.172
Mandible—Angle	9	0 (0)	1.022	0.312
Mandible—Ramus	10	3 (30)	4.417	0.036
Mandible—Condyle	1	0(0)	0	1
**<** **Hard tissue changes** **>**				
Defect in the trabecular bone	97	8 (8.2)	0.416	0.519
Osteosclerosis	34	2 (5.9)	0.695	0.405
Defect in the cortical bone	305	32 (10.5)	0.083	0.773
Sequestrum	354	38 (10.7)	0.28	0.597
Periosteal reaction	22	2 (9.1)	0.025	0.874

**Table 8:** Multivariate logistic regression analysis of recurrence-related risk factors.

Variable	OR	95% CI (LL–UL)	*p*-Value
Lesion site (ref = Maxilla)	Reference		
Mandible	1.356	0.645–2.85	0.422
Both jaws	4.555	1.542–13.46	0.006
Route of drug administration (ref = Oral)	Reference		
IV	0.501	0.287–0.872	0.015
Both	0.281	0.125–0.634	0.002
Systemic conditions (ref = no)	Reference		
Hyperlipidemia	0.398	0.169–0.935	0.034
Taking anticoagulants	0.255	0.06–1.087	0.065
Liver disease	3.703	1.079–12.711	0.037
Implant surgery	0.318	0.146–0.695	0.004
Affected area (ref = other area)	Reference		
Maxilla—Post. area	2.334	1.122–4.856	0.023
Mandible—Ramus	8.222	1.745–38.74	0.008
MRONJ Staging (ref = Stage 1)	Reference		
Stage 2	2.104	1.145–3.864	0.017
Stage 3	5.485	2.314–13.012	<0.001

## Data Availability

The original contributions presented in this study are included in the article. Further inquiries can be directed to the corresponding author.

## Abbreviations

The following abbreviations are used in this manuscript:

MRONJ	Medication-Related Osteonecrosis of the Jaw
IV	Intravenous
PO	Per Os (oral administration)
OR	Odds Ratio
CI	Confidence Interval
SD	Standard Deviation
